# Immune Response to the West Nile Virus in Aged Non-Human Primates

**DOI:** 10.1371/journal.pone.0015514

**Published:** 2010-12-02

**Authors:** Anne M. Wertheimer, Jennifer L. Uhrlaub, Alec Hirsch, Guruprasad Medigeshi, Jerald Sprague, Alfred Legasse, Jennifer Wilk, Clayton A. Wiley, Peter Didier, Robert B. Tesh, Kristy O. Murray, Michael K. Axthelm, Scott W. Wong, Janko Nikolich-Žugich

**Affiliations:** 1 Arizona Center on Aging, University of Arizona, Tucson, Arizona, United States of America; 2 Department of Immunobiology, University of Arizona, Tucson, Arizona, United States of America; 3 Vaccine and Gene Therapy Institute, Oregon Health and Science University, Beaverton, Oregon, United States of America; 4 Oregon National Primate Research Center, Oregon Health and Science University, Beaverton, Oregon, United States of America; 5 University of Pittsburgh Medical Center, Pittsburgh, Pennsylvania, United States of America; 6 Tulane National Primate Research Center, Covington, Louisiana, United States of America; 7 University of Texas Medical Branch, Galveston, Texas, United States of America; 8 University of Texas Health Science Center at Houston, School of Public Health, Houston, Texas, United States of America; Veterinary Laboratories Agency, United Kingdom

## Abstract

**Background:**

Risk of encephalitis from West Nile virus (WNV) infection increases dramatically with age. Understanding the basis of this susceptibility requires development of suitable animal models. Here, we investigated the immune response to WNV in old non-human primates.

**Methodology/Principal Findings:**

We investigated clinical, immunological and virological correlates of WNV infection in aging non-human primates. Aged (17–30yrs) and adult (6–9yrs) Rhesus macaques (RM) were challenged with WNV in the presence or the absence of the mosquito salivary gland extract (SGE) to approximate natural infection. None of the 26 animals exhibited clinical signs of the disease. Quantitative PCR suggested discrete and short-lived viremia, but infectious virus was never isolated. There was markedly increased, age-independent, proliferation of CD3^−^ non-B cells, followed by B-cell proliferation, which correlated to the loss of detectable WNV genomes. Moreover, animals primed with mosquito salivary gland extract exhibited reduced circulating WNV RNA. While we found the expected age-associated reduction in T cell proliferation, adaptive immunity did not correlate with infection outcome. That was further confirmed in a cohort of thymectomized and/or CD8 T-cell depleted Cynomolgus macaques (CM; N = 15), who also failed to develop WNV disease.

**Conclusions/significance:**

Results are consistent with strong and age-independent innate resistance of macaques against WNV challenge. This animal model is therefore not suitable for vaccine and therapeutic testing against WNV. However, understanding the basis of their innate resistance against WNV in macaques could provide helpful clues to improve anti-WNV protection of older adults.

## Introduction

West Nile virus (WNV) is a positive stranded RNA flavivirus, naturally transmitted in an enzootic cycle between mosquitoes and birds, which can readily infect a wide variety of dead-end hosts, including humans. It belongs to the Japanese encephalitis virus serocomplex of flaviviruses and causes human meningitic/encephalitic disease of varying severity.

WNV strain 1 clade a (1a) first entered the United States in Queens, NY, in 1999 spreading throughout the US by 2004 and providing an excellent example of a present day emerging pathogen. From 2004 to 2007 alone, CDC has registered >7800 cases of fever and >5000 cases of encephalitis in the US, with an approximate fatality rate of 10% following onset of encephalitis (www.cdc.gov/ncidod/dvbid/westnile/).

While 80% adults under the age of 50 experience no symptoms upon WNV infection, and only 1 in 150 experience severe disease with meningitis/encephalitis [1], [2], the situation is much more dire with advanced age. Lethality increases 10-fold in people over 50 and then to 40-50-fold at age 70, with a fatality rate of over 20% [3]. Despite intense efforts [4], [5] to date there is no approved human WNV vaccine. Treatment options remain partially effective, and recent reports suggest that current treatments may have no significant impact upon length of hospitalization [6]. Furthermore, the elderly are at greater risk of long term neurological defects from WNV infection, including chronic neurologic issues such as limb numbness or partial paralysis. Therefore, it is critical to understand protective immunity in adults and its decline in aging to devise appropriate vaccination strategies and immunomodulatory treatments to protect older adults against WNV [7].

Animal models have been invaluable in discerning key elements of susceptibility, persistence and resistance to strain 1a WNV [5], [8]. We showed in the mouse model that viral titers in the brains, but not in the blood and visceral organs, strictly correlated with mortality; WNV entered the brains of old and adult animals alike, but whereas most adult animals controlled neurovirulence, most old animals failed to do so [9]. This was due to profound defects in the development of antiviral effector CD4 and CD8 T cell response in old mice [9].

Rodent studies, however, do not always yield results that translate into humans, including failure in humans of vaccine approaches that were successful in mice [10]. Therefore, validation of immunological results in a non-human primate model is highly desirable. Prior work with adult Rhesus macaque (RM) exposed to infection with 10^5^ plaque-forming units (pfu) WNV found measurable viremia and humoral response, but no deaths nor clinical symptoms [11]. Another study found a clearly developed humoral response and a similar lack of clinical symptoms in baboons [12]. Finally, a natural outbreak of WNV at the Tulane NPRC, with over 700 animals exposed to WNV, also failed to reveal clinical symptoms or mortality [13]. One confounding issue in that study was the endemic exposure to flaviviruses in the area, which could not be controlled for in the natural experiment. To date, only direct intracranial infection of RM resulted in clinical presentation of WNV encephalitis [14] in non-human primates. As mentioned, the immunocompromised and the elderly have significantly increased risk of severe disease [1] associated pathology [15], and death. We therefore revisited the monkey model attempting to mirror natural human infection and included animals between 17–30 years of age (corresponding to 51–90 yrs in humans [16]) as well as immunodeficient animals, none of which were used in prior studies. We also included priming of the animals with mosquito salivary gland extracts (SGE), because recent mouse studies showed that this “natural” pretreatment may increase susceptibility to WNV [17]. Here we present our findings from several cohorts of adult, middle-aged and old RM and Cynomolgus (CM) macaques (n = 40), and conclude that macaques exhibit strong and age-independent resistance to this virus, that CD8 T cells are not required for protection and that resolution of infection likely involves robust innate immune mechanisms.

## Results

### Hypotheses, animal cohorts, dose and route of viral challenge

We tested three major hypotheses in this study: (1) That in aged animals, increasing the viral inoculum (based on prior studies) and/or altering the route of administration would produce neuroinvasion and clinical WNV disease (Cohorts 1 and 2); (2) That priming with mosquito salivary gland extract (SGE) prior and/or at the time of infection will increase susceptibility to infection (Cohorts 3, 4 and 5); and (3) That deficiency in cellular immunity will increase susceptibility of NHP to WNV disease (Cohort 5).

Cohorts 1 and 3–5 received 10^7^ pfu WNV, whereas cohort 2 received 10^9^ pfu WNV, as described in Table 1: Cohorts 1, 4 and 5 received the inoculum s.c., whereas cohorts 2 and 3 received a split injection of WNV i.v. and s.c. (80%:20% and 66%:34%, for cohorts 2 and 3, respectively). The rationale for split i.v. and s.c. route of infection was to mirror the natural mosquito feeding mechanism, where the mosquito probes and targets the capillary bed, while also depositing some of the virus, along with saliva, subcutaneously. For cohorts 1 and 2, a total of 5/7 aged and 3/5 adult animals were implanted with real time telemetry. Animals in cohorts 3–5 all received, in addition to the virus, the mosquito SGE, to mimic more closely natural infection, and also in the hope to produce a more pronounced clinical infection, as suggested by the results of SGE administration in rodents [17]. Cohort 3 (4 aged, 1 adult) and 4 (5 aged and 2 adult RM), with a total of 12 animals, were each primed with mosquito salivary gland extracts (SGE) prior to infection (Table 1). Of these, we implanted 7/9 aged and 3/3 adult monkeys with real time telemetry. Cohort 5 was set to test the effects of CD8 depletion with or without thymectomy (induced CD8 T-cell deficiency) upon resistance against WNV. This cohort contained 15 CM; 3 were CD8 depleted, 4 were thymectomized, 4 were thymectomized and CD8 depleted and the remaining 4 served as control animals. All were primed with SGE prior to s.c. infection and 9/15 were implanted with telemetry devices (Tab. 1).

**Table 1:** Rhesus macaque and Cynomolgus macaque cohorts.

Rhesus macaque cohorts								
Cohort	Adults (6–10) 7.6yr ave (age)		Aged(>17yrs) 21.5yr ave (age)		Total N	WNV dose	Priming with SGE (*Aedes albopictus*)	Route of administration
	Male	Female	Male	Female				
**1**	**2** (8,8)	**1** (6)	**2**(22,18)	**3**(20,19,18)	**8**	1×10(7) pfu/animal	N/A	subcutaneous
**2**	**2** (8,8)	**1**(10)	**2**(20,18)		**5**	1×10(9) pfu/animal	N/A	IV/subcutaneos (2 ml IV/500 ulSC)
**3**	**1** (6)		**1**(20)	**3**(30,21,20)	**5**	1×10(7) pfu/animal	Primed w/1 SGE at d-60, boosted at d-28, infected w/1 SGE at D0	IV/subcutaneous (1 mlIV/500 ulSC)
**4**	**2**(8,6)		**2**(25,20)	**3**(30,23,20)	**7**	1×10(7) pfu/animal	Primed w/1 SGE at d-60, boosted at d-28, infected w/1 SGE at D0	subcutaneous

Cynomolgus macaque cohorts								
Cohort	Sham		Thymectomized		N	WNV dose	Priming with SGE (*Aedes aegypti*)	Route of administration
	Not depleted (age)	ΔCD8 (age)	Not depleted (age)	ΔCD8 (age)				
**5**	**4** (14,13,13,13)	**3** (18,15,13)	**4** (15,13,13,11)	**4** (15,13,13,11)	**15**	1×10(7) pfu/animal	Primed twice a week w/1SGE final prime was at d(−)14 infect w/2 SGE at D0	subcutaneous

### Macaques exhibit no clinical symptoms directly attributable to WNV infection

All cohorts experienced changes in appetite and behavior during the acute phase post infection up to d14; however, due to the intense tissue sampling protocol these alterations could not definitively be ascribed to WNV infection. All cohorts maintained normal diurnal temperature rhythms (not shown). Two CM (Cohort 5) developed fever at day 7, coincident with elevated white cell counts, however, viremia was undetectable, and it was not possible to relate the fever with viral infection. Both resolved the fever and neither developed neurologic symptoms. In Cohort 5, 4/15 CM developed a localized rash at the site of administration of SGE, two of which subsequently developed a disseminated rash on their abdomen, inner arms and legs. This was concurrent with eosinophilia and likely represented an allergic reaction against mosquito saliva. Most importantly, no animals in any of the cohorts exhibited WNV-related neurological symptoms, regardless of age.

### Increased viral dose, intravenous route of infection and SGE priming correlate to reduced duration and levels of circulating viral RNA and the absence of viral shedding

**Figure 1:**
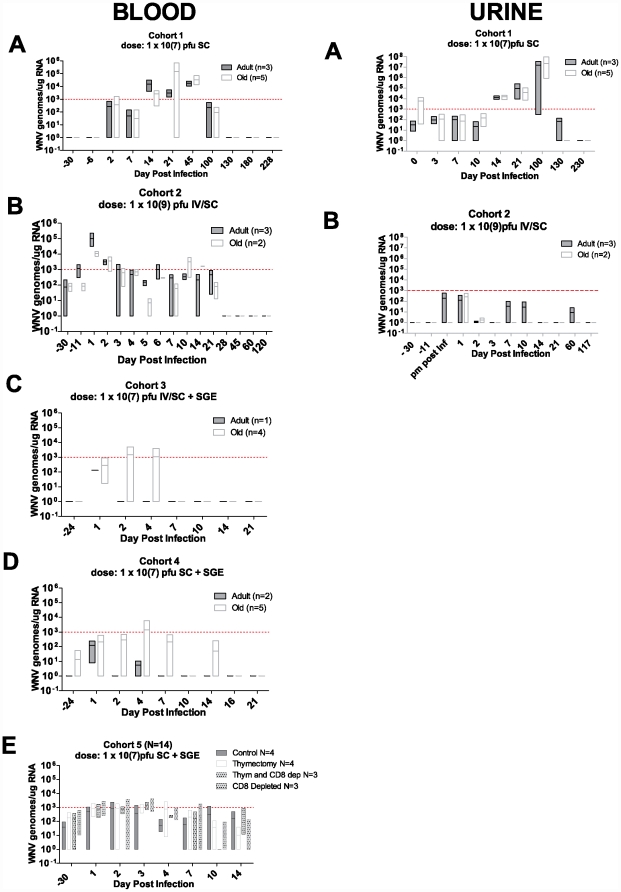
Increase of Viral Dose and Addition of Mosquito Salivary Gland Extracts (SGE) Decrease circulating Viral RNA. Quantitative PCR from each of the 5 cohorts as detected in Blood and Urine. Box plots illustrate maximum and minimum from each group. Bar illustrates population median. Red dashed line illustrates assay limit of detection. Blood Column titled Blood Panel A cohort 1, panel B cohort 2, animals primed with mosquito salivary gland extracts prior to infection are illustrated in Panel C cohort 3, panel D cohort 4, and panel E cohort 5. Column titled Urine Panel A and B illustrate the transient virurea detected only in cohort 1 (panel A), the cohort 2 had no detectable virus in the urine even though we examined time points as early as 5 hrs after IV infection.

Cohort 1 (1×10^7^ PFU/animal s.c.) exhibited surprisingly late presence of viral loads, from d14 until at least d45, peaking at day 21 (Figure 1A-Blood). There were no significant differences in viral titers or kinetics between the aged and adult animals (closed vs. open boxes). Cohort 2 (Figure 1B-Blood) received 100-fold higher WNV dose i.v. and s.c. Surprisingly, these animals had shown very brief presence of circulating viral genomes in all animals on d1 but disappearing by d4, and being significantly higher in the adult animals on d1. RM groups primed with SGE (Cohorts 3–5) also exhibited markedly decreased viral genome loads (Figure 1C–E), and also only between days 1–4. Although the peak levels of viral genomes observed in some animals were as high as 10^5^ genome equivalents/µg RNA, we were unable to culture WNV from plasma or whole blood in any of the animals in this study. In Cohort 1 and 2 each animal had detectable WNV RNA at some point (Cohort 1 = 8/8; cohort 2  = 4/4). In cohort 3, 4 and 5, 2/5, 2/7 and 12/15 animals had detectable WNV RNA, respectively This, together with lack of any clinical symptoms in any of the animals suggests that even when viral RNA was detected in the blood, its infectivity was greatly reduced.

We also evaluated viral shedding by examining urine (collected via cytesis, immediately stabilized in tri-reagent or flash frozen) from each cohort. We found detectable WNV RNA in cohort 1, where it appeared on day 14 and 21 in all 8 animals and was present at day 100 in 7 animals (Figure 1A-Urine). Again, we were unable to culture WNV even from the urine of animals exhibiting the highest levels of viral genomes. The viral genomes were detected by at least replicate assays, in both RNA stabilized and in flash frozen specimens, suggesting that detection was not an artifact linked to the method of sample preservation. No viral RNA was detected in the urine of any other cohort, suggesting that SGE presence (cohorts 3–5) as well as i.v. inoculation (cohorts 2 & 3) and/or higher viral dose (cohort 2) may stimulate earlier and more thorough viral clearance.

### Proliferation of leukocytes in RM upon WNV infection

**Figure 2:**
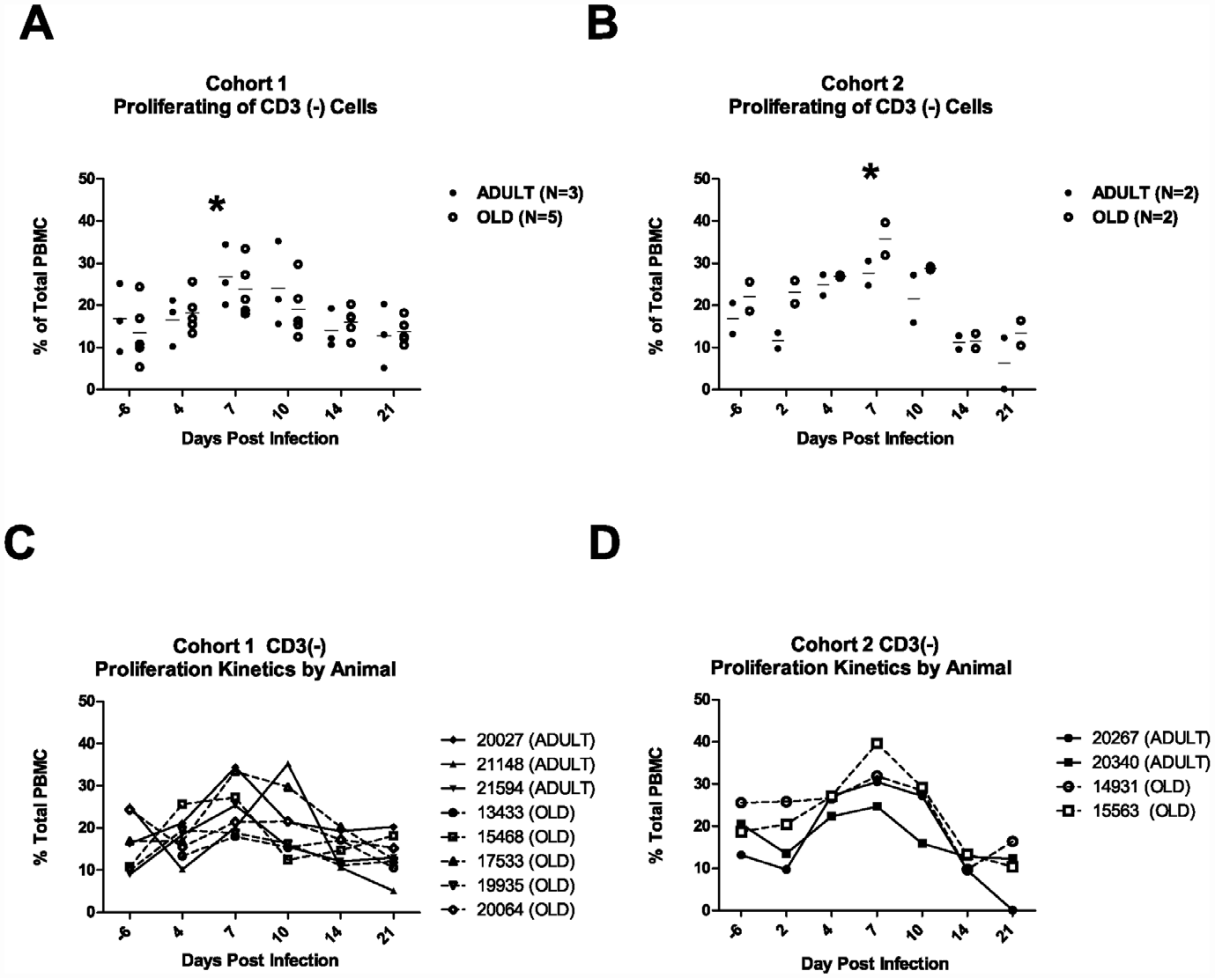
Proliferation of CD3- cells post WNV infection. The 4 panels illustrate kinetics of proliferation based upon levels of Ki67 measured in the total peripheral mononuclear cell population (PBMC) for cohort 1 and 2. Panels A and B illustrate proliferation of CD3 (−), non T cells, in cohort 1 and 2 respectively prior to infection through day 21. Each animal is represented by a dot (solid – adult; open - old). The center bar represents the mean. Panels C and D illustrate proliferation of CD3(−) cells within the total PBMC population for each animal. Dashed lines illustrate the aged animals solid lines illustrate the adult animals. All animals had increased proliferation of CD3(−), non-T cells, which peak by day 7 or 10 and then decline. Asterisks illustrate significant change (p>0.05).

**Figure 3:**
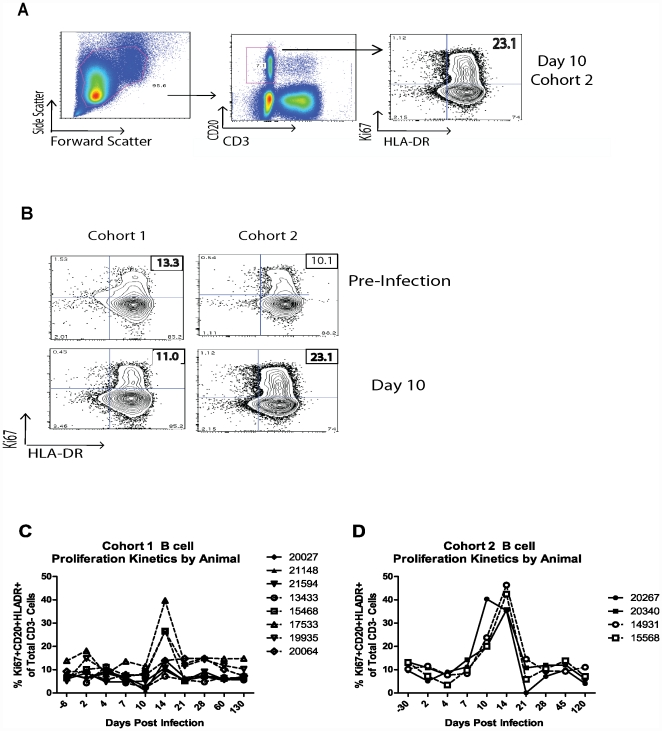
B cells Proliferate in Response to WNV Infection. Panel A and B: Representative profiles from flow cytometric analysis of PBMC from animals in Cohort 1 and Cohort 2 prior to and at day 10 post WNV infection. The first panel illustrates the physical size and cellular granularity (forward side scatter profile) of the PBMC. The next series of panels illustrate the sub populations defined first by expression of CD20 (to define B cells) and CD3 status (T cells vs non-T cells). The next series illustrates the proliferation (Ki67+) within the B cells (CD3- CD20+ HLA-DR+). The final calculations are expressed as percentages based upon the CD3- population for the B cells. Panel B illustrates the differences in B cell proliferation found in Group 1 vs. Group 2 at d10 post infection. Lower panels (C and D) illustrate B cell proliferation (CD3-CD20+HLA/DR+ based upon levels of Ki67 measured within the total CD3- populations for cohort 1 and 2. Panels C and D illustrate proliferation of Ki67+CD20+ cells in cohorts 1 and 2 respectively,. Dark lines illustrate the aged animals lighter lines illustrate the adult animals.

To characterize the immune response we investigated cellular phenotype and proliferation in PBMC from Cohorts 1 and 2. Proliferation was measured using Ki67, an intracellular marker which labels cells that traversed the S-phase of the cell cycle (past 2–3 days) in combination with various cell surface markers to identify T cells (CD3+) and B cells (CD3-, CD20+, HLA-DR+). Proliferating non-T cells (Ki-67^+^, CD3^−^) made up less than 20% (Figure 2A–B) pre-infection, but showed peak of proliferation between 30–40%, on Day 7 in both cohorts. Panels C and D illustrate the CD3^−^ cell proliferation kinetics for each animal over time in cohort 1 and 2 respectively. All animals exhibited marked proliferation of the non-T cell pool at day 7. Of note, no animals in Cohort 2 had detectable blood viral RNA at these time points (Day 4, 7, 10). We next sought to positively identify and examine proliferation of B cells. Figure 3A and B illustrates the gating strategy used to identify proliferation (Ki67^+^CD3^−^CD20^+^HLA-DR^+^) and frequency of B cells in Cohorts 1 and 2. B cell proliferated strongly in both cohorts but with a slightly later onset (d14 vs d10 Fig. 3C–D) and shorter duration in cohort 1; proliferation in both cohorts declined by day 21 (Figure 3C–D). Thus, B cells were responsible for d10–14 peak proliferation, but not for the d7 proliferation burst found in the CD3- population. There were no age-related differences in the extent of either non-B, CD3- or of the B-cell proliferation. Identity of the major proliferating day 7 cell population remains under investigation.

### Aged and adult macaques produce robust anti-WNV IgM and IgG antibody response

**Figure 4:**
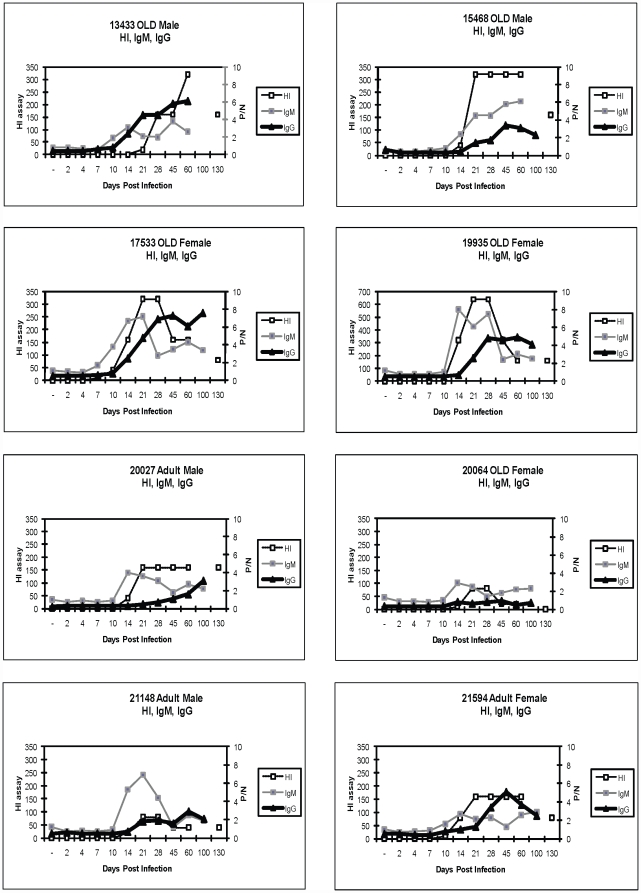
Aged and adult macaques produce robust anti WNV IgM and IgG response. Panels 1–8 illustrate WNV specific antibody production from Cohort 1 begins about day 10 peaking by day 21 and maintaining in most animals out well past day 45. HI (hemaglutination) assay on the left vertical axis, and IgG and IgM specific Elisa assays are on the right vertical axis reported P/N values (positive over negative), the assay limit of detection is P/N>2 is positive.

To assess whether B-cell proliferation was accompanied by production of protective antibodies, we measured total IgM and IgG anti-WNV Ab by ELISA, as well as their ability to inhibit hemagglutination (HI). Again, we observed no difference in Ab responses between old and adult animals. The majority of animals from cohort 1 made anti-WNV IgM antibody by d14, with a decline around d45, but often exhibited a second and persisting wave of IgM (Figure 4). Generally, IgG antibodies began to develop between d14 and 21 and persisted through the duration of the study. IgM was also detected in the CSF of 3 animals (1 old -17533) and 2 adults - 20027, 21148), either on d14 (17533), d28 (20027) or both (21148). In cohort 2, high dose infection (10^9^ pfu/i.v. and s.c.) was followed by an early rise in hemaglutinating antibody on d10, with a peak on d21 and a decline by d60 (not shown). Cohorts 3 and 4, primed with SGE (not shown) showed Ab production beginning on d14, and with all but one animal peaking by d28 and a decline by d60. Of note, the majority of animals in Cohort 1 (lower dose, s.c.), maintained their peak titer until d60, which correlated with prolonged circulating WNV RNA and its sporadic shedding. By contrast, animals primed with mosquito SGE (cohorts 3, 4 and 5) exhibited overall lower Ab titers, consistent with absent or barely detectable and transient WNV RNA. Fourteen of the fifteen animals from cohort 5 had measurable HI titers [18] on d30 or 60 post infection (not shown).

### Aged macaques exhibit delayed T-cell proliferative response to WNV infection adults

**Figure 5:**
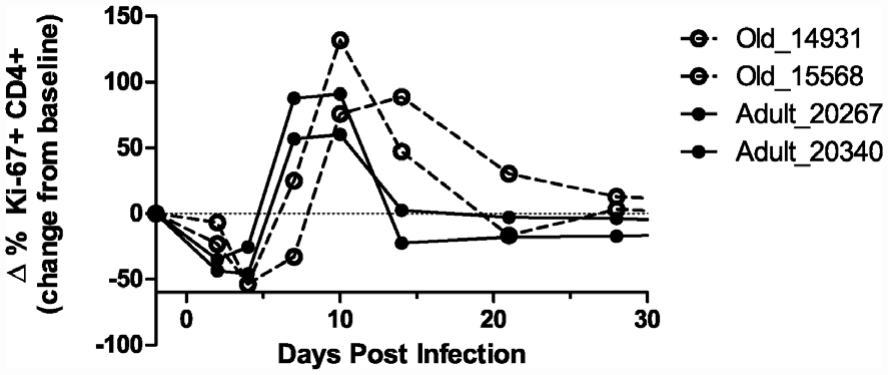
Analysis of cell cycle markers suggests an age associated delay in CD4+ proliferation. We used multi-parameter flow cytometry to evaluate the proliferation kinetics of CD4+ cells in each of our cohorts. The dark lines illustrate the two old animals whereas the lighter lines illustrate the two adult animals. The cell cycling is normalized to the baseline cycling recorded during this challenge for each animal, setting this to 0%. This illustration reveals that post WNV infection CD4+ cell cycling decreased followed by a marked increase on days 7, 10 and 14 which then wanes. Of particular interest is the age associated shift in kinetics both older animals (dashed lines) had a slower increase in CD4+ cycling than the adult animals (solid lines) and a prolonged phase prior to returning to a steady state post infection.

T cell proliferation (Figure 5) in the 4 animals from Cohort 2 provided additional important clues into the host-pathogen interaction with aging. This cohort clearly demonstrates that CD4+ T cells from old animals (open symbols with dotted lines) proliferate in response to WNV with delayed kinetics compared to adult RM, reminiscent of the results reported by Li et al. in a mouse tumor model [19]. However, this altered kinetic did not impact upon clinical disease, as no animals exhibited signs of WNV disease. All animals in this cohort displayed a prominent decrease in CD4+ proliferation on day 4, immediately prior to initiating the proliferative burst at about day 7. It is tempting to speculate that this is due to the withdrawal of all Ag-specific T-cells into the secondary lymphoid organs in the course of initial priming [20], however, additional experimentation is needed to address this issue conclusively.

### Lack of CD8 T-cells does not impair macaque resistance to WNV

**Figure 6:**
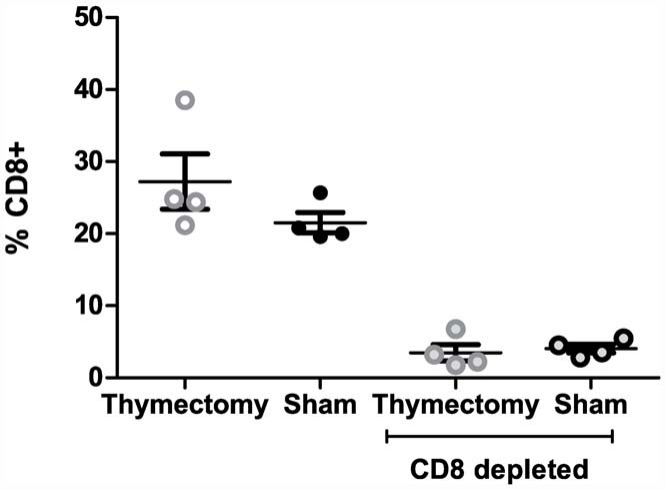
Immunodeficient animals – CD8 depleted animals survive WNV infection. CD8+ Tcells from previously thymectomized and CD8 depleted animals were enumerated from total PBMC using multi-parameter flow cytometry prior to WNV infection. Twelve months prior to infection animals were thymectomized (N = 4), thymectomized and CD8 depleted (N = 4), only CD8 depleted (N = 3) or controls (N = 4) (dark filled circles) Light circles illustrate thymectomized animals, dark circles illustrate control (sham operated) animals and light filled circles represent CD8 depletion.

Cohort 5 animal groups underwent thymectomy or CD8 depletion or both; the profound CD8 depletion in these animals is depicted in Figure 6. Despite this, none of these animals displayed neurological symptoms attributable to WNV infection. Viral load, as determined by qPCR in all the animals in this cohort was just above, but not significantly different from, baseline (via T test and Fishers exact test) (Figure 1E). Of note, all of these animals were primed with SGE prior to and at the time of infection and many of them displayed elevated eosinophiles. Clinical eosinophilia in some animals began 7 days after priming and continued after WNV infection. Levels of eosinophiles ranged from 1.2% (0.08×10^3^/ul) to 11.6% (0.83×10^3^/ul) [normal range 0–5% (0.0 to 0.4×10^3^/ul)] and varied from animal to animal regardless of their adaptive immune status (thymectomy or CD8 depletion). It is highly likely that this eosinophilia reflects reaction to SGE, and that reaction may well correlate with the rapid clearance of the virus, although mediators of this clearance remain to be identified.

## Discussion

In this study, we evaluated the effects of age, viral inoculum, route of infection, mosquito factors and immunocompetency on immunity against the WNV in the non-human primate model. We were unable to detect any clinical signs of the disease, despite the very advanced age of some of the animals, the use of CD8-depleted animals, and the very high dose of the virus inoculated. We confirmed prior reports that adult monkeys do not present with symptomatic WNV infection. More importantly, we here show that both aged and CD8-deficient non-human primates also resolve viral infection and fail to exhibit clinical symptoms of WNV disease when infected in a manner similar to the natural course of infection with a high dose of the virus. All animals displayed clear signs of contact with the virus, with measurable but quickly disappearing viral RNA, and/or by developing and maintaining humoral immune responses. In humans, only about 20% individuals develop symptoms, and 1∶150 ends up with severe, potentially fatal, encephalitis. The incidence of encephalitis, however, rises to 10% and higher in humans over 65 [1]. One of the significant limitations in our study is the small cohort size, and it could be argued that even the combined 40 animals may have not been sufficient to reveal the few animals which may present with clinical symptoms. However, at a face value, if macaque susceptibility was similar to that in humans, we should have observed symptoms in at least 8 animals. The fact that we used a substantial number of old animals, as well as animals with drastically reduced adaptive cellular immunity (which in humans and mice serves as strong predictor of susceptibility), allows us to conclude that sensitivity of old macaques to WNV is low, certainly lower than in mice (note, that the same WNV stock, lethal for most old B6 mice at 1,000 pfu - corresponding to ∼5×10^4^/kg - in our mouse studies [9] was used in the present macaque study without producing any clinical symptoms in monkeys) or humans. Therefore, we conclude that macaques are unlikely to provide a good model of age-related susceptibility to WNV, but may be useful to elucidate mechanisms by which they rapidly resolve WNV infection. In that regard, it should be noted that the various approaches employed to elicit a clinical response, including increased viral inoculum and priming with SGE, resulted in either a more rapid resolution of viral RNA loads, or a complete lack thereof, likely reflecting robust innate responses. Indeed, even when animals exhibited viral genomes in both the blood and urine, multiple attempts to culture infectious virus from urine, whole blood, or plasma were unsuccessful. Attempts to enrich for the virus from urine using co-cultivation in Vero cells were also unsuccessful (not shown). This could potentially occur due to natural antibody, complement neutralization (no preliminary evidence was found for either), or by other mechanisms, whose investigation is outside of the scope of this study. Paradoxically, we have noted the longest viral persistence (albeit coupled with the inability to culture infectious virus) in Cohort 1 animals, which received the relatively lowest viral dose in the absence of SGE. We speculate that this is due to the fact that in all other cohorts either higher viral doses or the presence of SGE probably stimulated stronger innate immune response, which promptly neutralized the virus. Consistent with this possibility, we found lower Ab responses in animals immunized in the presence of SGE, than in those not receiving the extract (Figure 4).

The humoral immune response in Cohort 1, which exhibited the most pronounced and longest viremia and WNV shedding, revealed a similar IgM response to Roehrig’s paper on persistence of IgM response in humans [21]. The human IgM response usually coincides with the onset of clinical symptoms. Normally this response declines over 2–3 months but we saw persistence of these responses, sometimes lasting up to day 230, and suggesting persistence of antigen. Also of interest was the presence of a secondary IgM wave of response. This data is compelling in light of results showing persistent WNV infection in hamsters [8] and findings of persistent IgM and non-culturable viral genomes shed in human urine [22]collectively suggesting that WNV may not behave as a typical acute pathogen. Whether the prolonged anti-WNV Ab response is needed to control persistent virus in macaques or whether it merely represents a bystander phenomenon, with other mechanisms keeping the virus at bay, remains to be elucidated. Of note, we did not observe differences between adult and old animals in the intensity of Ab responses. This could be interpreted to mean that in monkeys either the entire immune system or perhaps just B cells do not undergo the process of age-related deterioration seen in rodents and humans. We do not believe this to be correct, because B and T cells in RM readily exhibit the landmarks of immune senescence, including conversion into memory cells [23], naïve cell reduction [23], [24]and reduced T-cell effector function and Ab production in response to vaccination [25] and references therein]. Consistent with this, we observed a delay in T-cell proliferation in response to WNV infection, similar to the observations made in rodents [9] Rather, we believe it likely that the interaction between the specific virus or category of viruses and/or the high viral dose used in all animals may have circumvented any age-related differences and had produced a robust humoral response. As mentioned above, we do not believe that adaptive responses were necessary, because robust limiting of viral loads in SGE-immunized RM caused full protection, which was accompanied by low Ab titers in these same animals.

Our primary hypothesis was that including animals of significantly advanced age and increasing dosage or including intravenous route of infection would elicit neurological symptoms. Instead both of these parameters correlated to rapid resolution of circulating viral RNA. Our second hypothesis, that more severe WNV disease can be induced by priming animals with mosquito SGE (as would occur in WNV-endemic areas) and remarkably, our third hypothesis, that deficient antiviral response in CD8-depleted and thymectomized CM, will also increase severity of WNV disease, were also proven incorrect. It was recently [17] shown that increased mortality and reduced time to death occur when mice are pre-exposed to mosquito feeding. To mimic this paradigm we administered SGE in micro-subcutaneous injections but found instead enhanced protection, as evidence by the decreased viral load (including 20/20 aged and immunocompromised animals). This raises interesting questions on protection vs. exacerbation of the vector-borne disease. Our current study provides suggestive evidence of a heightened innate immune response including elevation of total monocyte counts (i.v. infection, not shown) and prolonged eosinophilia, in animals primed with SGE, however, these leads will require additional work to establish their importance. Indeed, understanding the mechanisms of protection at play in old and immunocompromised macaques against WNV could provide new therapeutic targets and strategies against this and other similar viral infection in the old age.

## Materials and Methods

### Animals

Colony bred and wild caught male and female Rhesus macaques (*Macaca Mulatta*) (RM) and Cynomolgus macaques (*Macaca Fascicularis*) (CM) were maintained according to federal, state and local guidelines. All RM were of Indian origin, whereas CM were from China and Mauritius. Nonhuman primate studies were performed in biosafety level 3 biocontainment at the Oregon national Primate Research Center (ONPRC) and the protocol was approved by the Oregon Health Sciences’ ONPRC Animal Care and use Committee and carried out in strict accordance with the recommendations of the National Institutes of Health’s (NIH) “Guide for the Care and use of Laboratory Animals” and the U.S. Animal Welfare Act. The ONPRC is an American Association for Accreditation of Laboratory Animal Care (AAALAC)-accredited, NIH-supported non-human primate (NHP) research facility and has an approved Assurance (#3304-01) for the care and use of animals on file with the Office for Protection from Research Risks at NIH. Animals were acclimatized for 21 days prior to infection. Animals were monitored (pre- and post-infection) and fed commercial monkey chow, treats and fruit twice daily. Environmental enrichment consisted of commercial toys and visual stimulation. All surgical and invasive clinical procedures were conducted by trained personnel under the supervision of veterinarians in dedicated surgical facilities using aseptic techniques and comprehensive physiologic monitoring. Ketamine HCl was used to induce anesthesia for all routine non-invasive clinical procedures associated with the study protocol such as blood sampling, bronchoalveolar lavage, drug administration and clinical examinations or treatment. Telazol® was used to induce anesthesia for peripheral lymph node biopsy. All animals were provided analgesia during and after potentially painful procedures. Animals were pre-screened to be free from tumors, signs of clinical disease, and antibodies against West Nile and other related flaviviruses (SLEV, JEV. TBE, YFV, DEN1-4). Cohort description (25 RM and 15 CM) is shown in Table 1.

### Infection and sampling of macaques

The WNV viral stock was derived from the second-round passage of a snowy owl liver isolate (WNV 385-99) grown in C6/36 insect cells, followed by sucrose gradient purification. The same viral stock was used for all experimental infections, and dose verified by plaque assay using the material left over after infection. Doses and routes are described in Table 1. Mosquito salivary gland extract (SGE), one (1) SGE was defined as the sonicate from one individual mosquito’s pair of salivary glands. Animals were sampled by lymph node biopsy, bronchio-alveolar lavage, cerebrospinal fluid (CSF), urine (via cytesis) and blood for baseline measurements prior to infection or mosquito salivary gland priming and sampled post infection at indicated intervals from indicated tissues.

### Telemetry

Telemetry was used to acquire in-vivo temperature & activity every 2-5′, 24 h/day; it consisted of implantable TA-D70 transmitters and receivers (Data Sciences International, St. Paul, MN), data matrix, PCI card, and VitalView software (Mini Mitter, Bend, OR). Data were processed using MSExcel, and OriginLab graphing software was used to provide visual representations of change over time.

### Reverse Transcription quantitative PCR (qPCR)

One step RT-PCR kit [master mix and RT; Applied Biosystems (Taqman, One-step RT-PCR master mix reagents, cat # 4309169)] was used to amplify a 100 bp segment of the WNV genome within the NS3 region [26] [primers: Fwd: 5′-GCACTGAGA GGACTGCCCAT Rev: 5 -TGGGTGAGGGTAGCATGACA Probe: 5 -6FAM-TACCAG ACATCCGCAGTGCCCAGA-T-TAMRA] were used for 40 cycles of: 48°C, 30′; 95°C, 10’; 95°C, 15′′→60°C 1′. Assay limit of detection was set at 1000 genome copies/µg RNA.

### Flow Cytometry

PBMCs were stained for phenotype and proliferative response as in [23], [24] using mAb against human CD3, CD4, CD8? (Beckman Coulter, clone 2ST8.5H7),CD20, and HLA-DR, (BD, eBioscience and BioLegend). Following washing, fixing, permeabilization, and overnight DNA digestion (0.28 mg bovine pancreas-derived DNase, Sigma–Aldrich), intracellular staining was performed using anti-Ki-67-FITC (Becton Dickinson, clone B56) for 30’. At least 3×10^5^ events were acquired using the LSR-II cytometer (Becton Dickinson) equipped with DiVa software and the FCS3.0 file system, and analyzed using FlowJo 8.2 (TreeStar) software.

### Hemaggluination-inhibition (HI) assays

HI antibodies to WNV antigen were measured as previously described [18]. Plasma was screened at 1∶10 against WNV and SLEV antigens. Positive samples were subsequently titrated using serial 2-fold dilutions.

### Anti-WNV IgG and IgM ELISA

The CDC protocol for IgG and IgM antibody capture enzyme-linked immunosorbent assay (MAC-ELISA) was followed [27]. Briefly, we used duplicate samples with positive and negative controls. An average absorbance of each was determined at 450 nm. The optical density of the experimental plasma was divided by that of the negative control to determine the P/N ratio. Test interpretation was as follows: P/N<2 = negative result, P/N = 2 to 3 = equivocal result, P/N>3 = positive.
